# Oral Toxicity of *Pseudomonas protegens* against Muscoid Flies

**DOI:** 10.3390/toxins13110772

**Published:** 2021-11-01

**Authors:** Luca Ruiu, Maria Elena Mura

**Affiliations:** Dipartimento di Agraria, University of Sassari, 07100 Sassari, Italy; mariaelenamura@uniss.it

**Keywords:** entomopathogens, microbial, biological control, insecticidal genes, virulence factors, pest management, mode of action

## Abstract

The bioinsecticidal action of *Pseudomonas protegens* has so far been reported against some target insects, and the mode of action remains unclear. In this study, the pathogenicity potential of a recently isolated strain of this bacterial species against fly larvae of medical and veterinary interest was determined. Preliminary experiments were conducted to determine the biocidal action by ingestion against *Musca domestica* and *Lucilia caesar* larvae, which highlighted a concentration-dependent effect, with LC_50_ values of 3.6 and 2.5 × 10^8^ CFU/mL, respectively. Bacterial septicaemia was observed in the body of insects assuming bacterial cells by ingestion. Such rapid bacterial reproduction in the hemolymph supports a toxin-mediated mechanism of action involving the intestinal barrier overcoming. In order to gain more information on the interaction with the host, the relative time-course expression of selected *P. protegens* genes associated with virulence and pathogenicity, was determined by qPCR at the gut level during the first infection stage. Among target genes, *chitinase D* was the most expressed, followed by *pesticin* and the fluorescent insecticidal toxin *fitD*. According to our observations and to the diversity of metabolites *P. protegens* produces, the pathogenic interaction this bacterium can establish with different targets appears to be complex and multifactorial.

## 1. Introduction

*Pseudomonas protegens* is a soil-dwelling bacterium characterized by an increasing scientific and industrial interest as a growth promoting agent for cultivated plants [1]. Like other pseudomonad species, this bacterium is able to colonize the rhizosphere favoring biochemical mechanisms increasing the availability of soil nutrients to the plant. In addition, pseudomonads can act indirectly (plant resistance induction) and directly (i.e., antibiosis) against phytopathogens [2]. The direct action against plant pathogens relies on the production of diverse compounds such as 2,4-diacetylphloroglucinol (DAPG), hydrogen cyanide (HCN), orfamide A, pyoluteorin, pyrrolnitrin, rhizoxin derivatives, toxoflavin, and several enzymes [3]. While some of these metabolites may also have a toxic or inhibitory action against insect pests affecting crops, several *Pseudomonas* species have developed particular toxins and virulence factors to act more specifically against insects. One of the most studied members of this bacterial genus is *P. entomophila* that acts by ingestion against susceptible targets leveraging a specific toxin secretion system [4]. Similarly, diverse species in the *P. fluorescens* group may act as insect pathogens [5]. However, those belonging to the sub-clade 1, including *P. protegens*, seems to have developed specific gene traits associated with the insecticidal properties [6]. Recent studies have documented an oral infection process implying a fluorescent insecticidal toxin (Fit) complex showing similarity with the makes caterpillars floppy (Mcf) toxin found in the entomopathogenic nematode symbiont *Photorhabdus luminescens* [7]. However, *P. protegens* mutants lacking the functionality of the gene complex *fit* were observed to be still active against fruit flies, thus supporting the involvement of other insecticidal compounds [8]. In the light of the few studies available, the mechanism of action of *P. protegens* on susceptible insects still appears to be poorly understood. In order to increase understanding of *P. protegens* target range and entomopathogenic mode of action, we conducted bioassays on different muscoid fly larvae investigating the possible involvement of genes encoding for some putative protein toxins and virulence factors. For this purpose, gene expression profile was studied at the transcriptional level after oral administration of a new strain of the bacterium. Because target genes are in common between different *P. protegens* strains, the output of this study is useful to deepen the knowledge also of other strains of this species.

## 2. Results

Different experiments were designed to investigate the action of *P. protegens* on larvae of two common muscoid fly species, the housefly *Musca domestica* L. and the blow fly *Lucilia caesar* L. For this purpose, the recently isolated strain CO1, whose insecticidal properties against a wide range of pests was previously reported [9], was used.

### 2.1. Oral Toxicity

To determine the ability of *P. protegens* to act in the gut of larvae after ingestion, insects were reared for 72 h on artificial diets incorporating different concentrations of bacterial cells. As a result, the bacterium was found to have a clear toxic action on both house fly and blow fly larvae, which confirms its ability to interact with gut barriers (i.e., peritrophic matrix, epithelium) and, eventually, cause a septicaemia.

**Figure 1:**
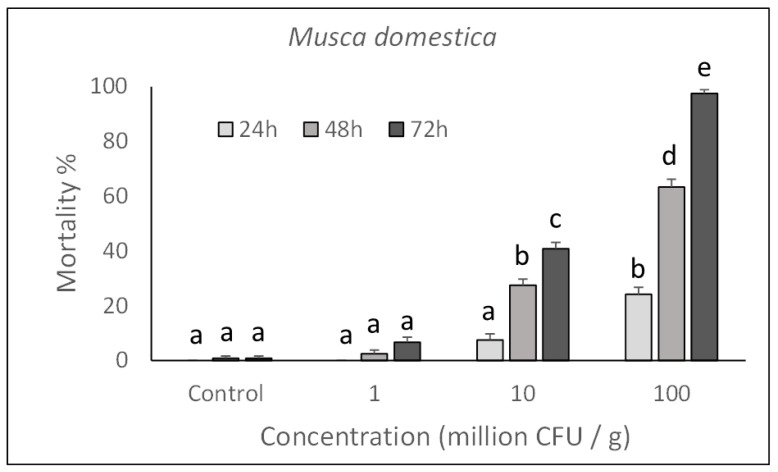
Over time mortality (mean ± SE) of *Musca domestica* larvae reared on a diet treated with different *P. protegens* concentrations. Different letters above bars indicate significantly different means (ANOVA Mixed Proc., Tukey adjusted *p* < 0.05).

**Figure 2:**
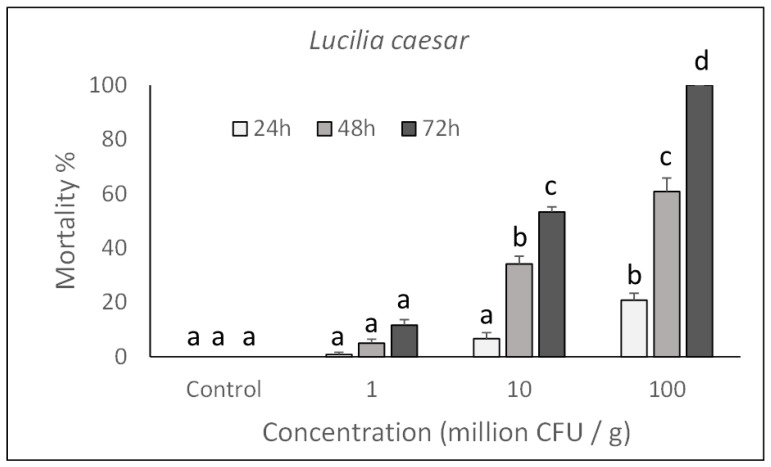
Over time mortality (mean ± SE) of *Lucilia caesar* larvae reared on a diet treated with different *P. protegens* concentrations. Different letters above bars indicate significantly different means (ANOVA Mixed Proc., Tukey adjusted *p* < 0.05).

As shown in Figure 1 and Figure 2, toxicity was concentration-dependent and a significant action with 100% mortality for the highest concentrations assayed, was achieved on both species after rearing larvae for 72 h in a treated diet (*M. domestica*: F_3,132_ = 760.74, *p* < 0.0001; *L. caesar*: F_3,132_ = 493.72, *p* < 0.0001). Significant increase in mortality was observed in samples exposed for a longer time to each assayed concentration (*M. domestica*: F_2,132_ = 258.85, *p* < 0.0001; *L. caesar*: F_2,132_ = 251.21, *p* < 0.0001).

Based on the Probit analysis, *M. domestica* and *L. caesar* larvae appeared to have a similar susceptibility to *P. protegens* with an LC_50_ (CI) value of 3.6 (2.1–4.9) × 10^8^ CFU/mL for *M. domestica* (Slope = 1.872 ± 0.19; χ^2^ = 14.16; df = 86) and 2.5 (1.1–4.1) × 10^8^ CFU/mL for *L. caesar* (Slope = 2.804 ± 0.26; χ^2^ = 19.63; df = 86)).

A bacterial septicaemia was observed in hemolymph samples collected from dead larvae and observed under a phase microscope.

### 2.2. Post-Injection Pathogenicity

**Figure 3:**
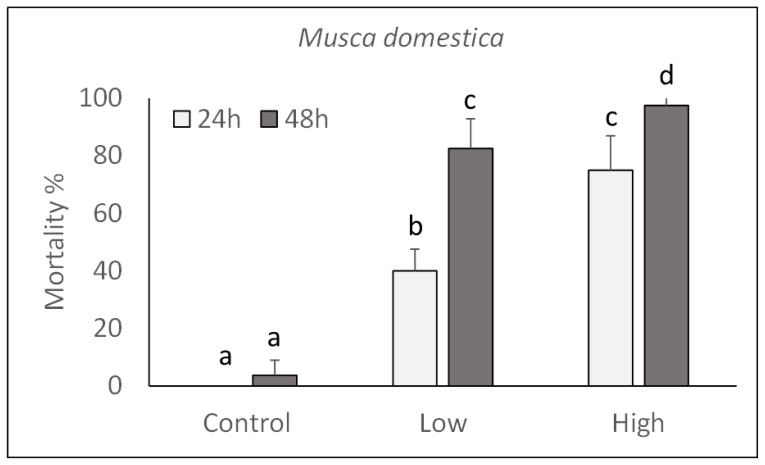
Over time mortality (mean ± SE) of *Musca domestica* larvae injected with different doses of *P. protegens* (high = 1000 CFU/larva; low = 100 CFU/larva). Different letters above bars indicate significantly different means (ANOVA Mixed Proc., Tukey adjusted *p* < 0.05).

**Figure 4:**
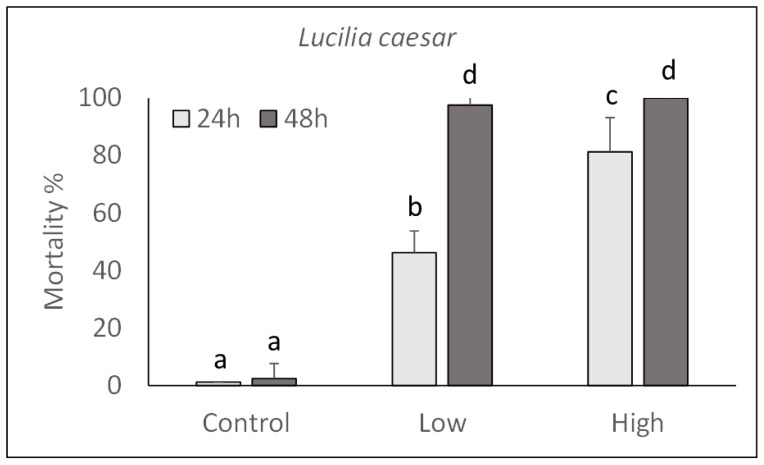
Over time mortality (mean ± SE) of *Lucilia caesar* larvae injected with different doses of *P. protegens* (high = 1000 CFU/larva; low = 100 CFU/larva). Different letters above bars indicate significantly different means (ANOVA Mixed Proc., Tukey adjusted *p* < 0.05).

The pathogenicity of *P. protegens* strain CO1 was evaluated on *M. domestica* and *L. caesar* larvae by injecting two doses (high: 1000 CFU/larva; low: 100 CFU/larva) into their body and assessing their mortality during the next 48 h. As shown in Figure 3 and Figure 4, a significant increase in mortality percentage nearly reaching 100% in 48 h for both fly species, was observed in comparison with control larvae injected with PBS. Mortality was significantly affected by the injection dose (*M. domestica*: F_2,47_ = 469.65, *p* < 0.0001; *L. caesar*: F_2,47_ = 884.42, *p* < 0.0001), time after injection (*M. domestica*: F_1,47_ = 98.49, *p* < 0.0001; *L. caesar*: F_1,47_ = 171.00, *p* < 0.0001) and the interaction between these factors (*M. domestica*: F_2,47_ = 23.47, *p* < 0.0001; *L. caesar*: F_2,47_ = 65.05, *p* < 0.0001).

**Figure 5:**
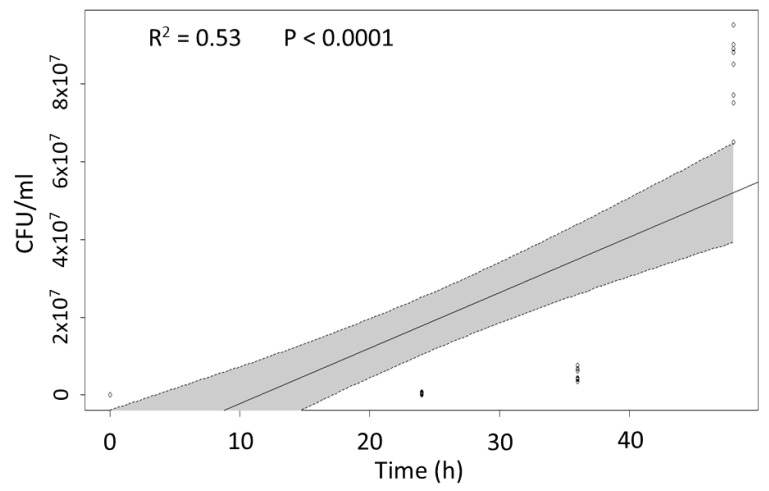
Linear regression plot with 95% confidence intervals (shaded areas) representing the relationship between *P. protegens* abundance in the insect body (CFU/mL of hemolymph) and time after bacterial inoculation.

In order to determine the ability of *P. protegens* to reproduce in the insect haemocoel, the time-course bacterial growth was determined in hemolymph samples collected from *M. domestica* larvae injected with 1000 CFU. A progressive and rapid increase in bacterial abundance in the larval body was observed at consecutive time intervals after injection, confirming the suitability of insect tissues for bacterial growth. A significant correlation between concentration and time was therefore observed (adjusted R^2^ = 0.5273, F = 44.5, *p* < 0.0001) (Figure 5).

### 2.3. In Vivo Expression of Bacterial Genes

**Figure 6:**
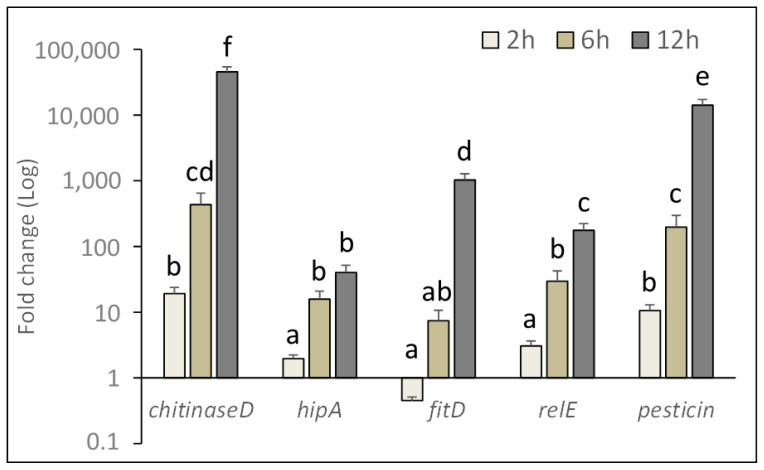
Relative expression of pathogenicity related genes of *P. protegens* at the gut level of treated house fly larvae during the initial infection phase. Fold change was calculated using bacterial *16S rRNA* and insect *β-actin* transcript abundance for qPCR data normalization. Different letters above bars indicate significantly different means (2-ways ANOVA, Tukey adjusted *p* < 0.05).

Based on the lethal effects observed in ingestion bioassays and on the ability of *P. protegens* to reproduce in the insect body, gene expression analyses were conducted to gain new knowledge about the mechanism of action enacted by the bacterium during the first phase of infection. For this purpose, relative time course expression of *P. protegens* genes associated with virulence and pathogenicity, was determined at the gut level during the first infection stage. Relative expression of selected target genes (*chitinase D,* *toxin HipA, toxin RelE, pesticin, cytotoxin FitD*) at different time intervals (2, 6 and 12 h) after exposing house fly larvae to bacterial cells is shown in Figure 6.

Target gene (F_4,134_ = 23.16, *p* < 0.0001), time after injecting the bacterial inoculum (F_2,134_ = 43.48, *p* < 0.0001), and the interaction of these two factors (F_8,134_ = 22.50, *p* < 0.0001), significantly affected gene expression level in the insect gut.

A significant increase in the relative expression of these genes was observed over time, achieving a maximum at 12 h after exposure to the bacterium, (*chiD*: F_3,29_ = 56.35, *p* < 0.0001; *hipA*: F_3,29_ = 22.11, *p* < 0.0001; *fitD*: F_3,29_ = 61.90, *p* < 0.0001; *relE*: F_3,29_ = 38.37, *p* < 0.0001; *pesticin*: F_3,29_ = 49.28, *p* < 0.0001). Among target genes, *chitinase D* was the most expressed, followed by *pesticin* and *fitD*.

## 3. Discussion

The genus *Pseudomonas* includes several species with insecticidal properties, such as the well-known *P. entomophila* that acts by ingestion causing damages to the midgut epithelium of susceptible insects, exploiting a specific toxin secretion system [4]. Accordingly, genomic analyses of different pseudomonads have revealed some well-conserved insecticidal traits, in particular associated with the *P. fluorescens* group that, within clade-1, includes *P. protegens*. Consistently, our study demonstrated that strain CO1 of *P. protegens*, originally isolated from the hemolymph of diseased *G. mellonella* larvae [9], is toxic by ingestion to *M. domestica* and *L. caesar* larvae. Such results, corroborate previous reports on the *per-os* insecticidal potential of this bacterial species, supporting the ability of the bacterium to interact at the intestinal level [10]. Lethal effects were concentration-dependent, as normally expected for entomopathogenic bacteria acting by ingestion on these target insects [11]. LC_50_ values are comparable with those observed on other entomopathogenic bacteria with similar mode of action and effective on muscoid flies [12]. Our experiments conducted injecting bacterial cells in the house fly larvae haemocoel, showed that *P. protegens* reproduces well in the hemolymph, which represents a natural target substrate ultimately allowing the bacterium to express its biotic potential. However, when bacterial cells enter the insect body by ingestion, in order to reach the haemocoel, the intestinal barriers, mainly represented by the peritrophic matrix and the epithelium, have first to be overcome.

Similar to other entomopathogenic bacterial species active by ingestion, such as the well-known *Bacillus thuringiensis*, the pathogenic process is expected to involve insecticidal toxins interacting specifically with the intestinal epithelium. Normally, this interaction involves the binding to epithelial cell receptors and the alteration of cell permeability with a consequent flux of ions and water, leading to the intestinal barrier disruption and allowing the bacterium to reach the hemocoel [13]. According to such scenario, in our ingestion experiments, dead larvae showed a bacterial septicaemia. To achieve this, the bacterium must be equipped with an adequate arsenal of toxins and virulence factors. Consistently, *P. protegens* was observed to harbor some insecticidal-related genes ([6], like other members of the *P. fluorescens* group, in which insecticidal toxin complex (Tc) gene homologues typical of the entomopathogenic nematode symbiont *Xenorhabdus* and *Photorhabdus* species, are frequently found [14]. In the case of *P. protegens*, the insecticidal potential has been attributed to the fluorescent insecticidal toxin (Fit), a large protein similar to the Makes caterpillars floppy (Mcf) toxin produced by *Photorhabdus luminescens* [7]. The *fitD* gene domain is flanked by a protein secretion system including *fitABC* and *fitE* genes and the regulatory genes *fitG* and *fitH*. While the expression of *fitD* gene after bacterial injection or ingestion was shown to be involved in the insecticidal action, significant *per os* toxicity of *P. protegens* mutants lacking *fitD* gene was observed in *Drosophila melanogaster*, which supported a more complex mechanism of action involving other virulence factors, among which orfamide A, chitinases and phospholipases were reported as possible candidates [6,8]. Although the implication of some of these compounds in the insecticidal action has been at least partially documented, numerous aspects of the mechanism of action remain unclear.

Our study with house fly larvae investigated the possible role of a selection of genes, well conserved in the genome of *P. protegens*, in the initial phase of infection, following ingestion of bacterial cells. The overtime increasing over-expression of these genes, supports their involvement in an action against midgut barriers, which would precede haemocoel invasion and septicemia. In addition to FitD that might be directly involved in cytotoxicity toward epithelial cells [15,16], the observed upregulation of *chitinase D* may significantly help overcoming the first barrier offered by the peritrophic matrix containing chitin. This hypothesis corroborates the mode of action observed in Tc-like protein complexes containing eighter insecticidal toxins and chitinases acting synergistically to break through the intestinal barrier [17]. The engagement of other putative virulence factors such as *pesticine*, *hipA* and *relE* may further increase *P. protegens* virulence and help counteracting the innate immune response that normally comes into action at the gut level of insects challenged with an entomopathogenic bacterium to which they are susceptible [18]. The same and other virulence factors expressed by *P. protegens* might later be involved in favoring the successive stages of the pathogenic process within the insect body. A wider overview of the bacterial metabolites involved in pathogenesis against muscoid flies would be provided by a broader transcriptomic analysis involving RNA-seq analyses.

According to our observations and to the discovery of several metabolites *P. protegens* may produce, the pathogenic interaction this bacterium can establish with different targets appear to be complex and multifactorial [16]. Evidence in our study of the biocidal potential against pests of veterinary importance, provides additional biological information to the previously reported bioinsecticidal action this bacterial species shows against crop pests and its plasticity establishing plant-beneficial interactions [19]. Despite different degrees of virulence are expected to be associated with diverse *P. protegens* strains, the maintenance within the species of an arsenal of toxins and virulence factors, suggests an evolutionary process in which a conserved pathogenic relationship with insects became established. Future studies will clarify the target range and the role of specific bacterial virulence factors in pathogenesis.

## 4. Materials and Methods

### 4.1. Bacterial Strain and Preparations

*Pseudomonas protegens* strain CO1 from the collection of the University of Sassari was selected for this study because of its previously determined toxicity against fly larvae [9]. Bacterial cells were cultured for 72 h in Luria–Bertani (LB) broth at 30 °C with shaking at 180 rpm. After being harvested by centrifugation at 10,000 rpm for 10 min, cells were resuspended in PBS and quantified through serial dilutions on LB agar plates to determine the number of CFU/mL. Fresh suspensions were adjusted with water or PBS to obtain the concentration needed in each bioassay.

### 4.2. Bioassays

Bioassays were conducted with larvae of *Musca domestica* L. (Diptera: Muscidae) and *Lucilia caesar* L. (Diptera: Calliphoridae) from colonies maintained at the insect laboratory of the Dipartimento di Agraria of the University of Sassari (Italy) using the methods of Ruiu et al. [20].

#### 4.2.1. Ingestion Bioassays

To assess the *per os* toxicity of *P. protegens* on target insects, second instar larvae of *M. domestica* or *L. caesar* were reared on a diet incorporating the bacterial cell suspension (vigorously mixed with a spatula) at a specific concentration or left untreated (control), according to methods described in Ruiu et al. [9]. Larvae were maintained inside a growth chamber at 25 °C and 60% R.H, in groups of 10 individuals inside Petri dishes (3.5 diameter) containing an artificial diet (2 g) made of wheat bran (34%), milk powder (1%), and water (65%) (wt/wt) for *M. domestica*, and moistened dog food for *L. caesar*. Each experiment involved 4 replications and was repeated 3 times. Mortality was recorded daily for 72 h. To determine the median lethal concentration, the following concentration range was assayed: 1 × 10^9^, 5 × 10^8^, 2.5 × 10^8^, 1 × 10^8^, 5 × 10^7^, 2.5 × 10^7^, 1 × 10^7^, 1 × 10^6^ CFU/g of diet.

#### 4.2.2. Injection Bioassays

In order to determine the ability of *P. protegens* to reproduce in insect haemocoel, bacterial cell suspensions were injected by a Hamilton syringe (2 µL/larva) into the ventral intersegmental region of surface sterilized third instar larvae of *M. domestica* and of *L. caesar*. Each larva received either a higher (1000 CFU/larva) or a lower (100 CFU/larva) dose. Control larvae were instead injected a PBS solution. Infected and control larvae were maintained on filter paper at 25 °C and 50% R.H. inside Petri dishes in groups of 10 individuals. Each treatment had four replicates and larvae were inspected every day for 48 h to assess mortality.

In a different injection experiment according to the same design and involving 3 replicates, pools of 10 larvae were sampled at different time intervals after injection (12, 24, 36, and 48 h) and their hemolymph collected to be analyzed for the abundance of *P. protegens*. For this purpose, the number of colony forming units (CFU) was determined by serial dilutions and growth on LB plates. This experiment was repeated twice with three technical replicates.

### 4.3. Insecticidal Protein Gene Expression at Gut Level

This experiment was designed to investigate the involvement of bacterial virulence factors in the post-ingestion action at the gut level. For this purpose, a selection of pathogenicity related genes was preliminarily identified in the genome of reference *P. protegens* strain CHAO, taking into account previous reports on this bacterial species virulence against insects [15,16,19]. This included genes encoding for chitinases and insecticidal toxins as listed in Table 1 that shows primer pairs designed on their sequences for qPCR. Expression of these genes was determined at the transcriptional level on pools of *M. domestica* larvae collected at different time intervals (2, 6, 12 h) after being reared on the previously described artificial diet incorporating *P. protegens* at a concentration of 10^8^ CFU/g. Individually dissected intestines from treated and untreated (control) larvae were pooled (*n* = 10) for total RNA extraction using TRIzol^®^ Reagent (Life Technologies, Carlsbad, CA, USA) following manufacturer’s protocol [21]. After being quantified and purity checked by a NanoDrop ND-1000 Spectrophotemeter (Thermo Scientific, Waltham, MA, USA), RNA was treated with RQ1 RNase-Free DNase (Promega) and retrotranscribed to first-strand cDNA with Random Hexamer Primers (Life Technologies), SuperScript^®^ II Reverse Transcriptase (Life Technologies) and RNaseOUT™ Recombinant Ribonuclease Inhibitor (Life Technologies) in accordance with manufacturers’ instructions. Quantitative PCR reactions were run with Power SYBR^®^ Green PCR Master Mix (Life Technologies) on an Applied Biosystems 7900HT Fast Real-Time PCR System using the following cycle conditions: denaturation at 95 °C for 10 min, followed by 40 cycles of 95 °C for 15 s, annealing at 58 °C for 1 min, and extension at 60 °C for 1 min. Primer pairs used in qPCR (Table 1) were preliminarily tested by standard curve and dissociation curve analyses [22] and transcripts abundance was determined according to Livak and Schmittgen [23] using *16 rRNA* as *P. protegens* internal control gene and *β-actin* as *M. domestica* reference gene for PCR normalization [18]. Three biological replicates (10 flies each) were considered for each analysis that involved three technical replicates.

### 4.4. Statistical Analysis

Statistical analyses were performed with R software version 3.10 [24] with significance level set at α = 0.05.

Repeated measures ANOVA (PROC MIXED) with means separation using LSMEANS comparison (adjust = Tukey), was used to analyze overtime mortality data.

The relationship between time and bacterial septicaemia (CFU) in the insect body, was analyzed by linear regression analyses, while median lethal concentrations (LC_50_) were calculated by probit regression.

Two-ways ANOVA, followed by multiple comparison of means (adjust = Tukey) was used to analyzed gene expression fold changes.

## Figures and Tables

**Table 1:** Oligonucleotide sequences used for qPCR analyses.

Toxin Gene	Abbreviation	Acc. Number	Primer Sequence	
			Sense 5′-3′	Antisense 5′-3′
*chitinase D*	*ChiD*	NC_021237.1	5′ CATATCGAATTGCACAAGGGCAACGAACAG 3′	5′ AGGCGCCATGCTGATGATGAAGTGCTT 3′
*toxin HipA*	*HipA*	NC_021237.1	5′ CTGCGACATGCTCAGAAGCGAGTTCCACTA 3′	5′ ACGCAGGTAGTCGGCCACCAGCTC 3′
*toxin RelE*	*RelE*	NC_021237.1	5′ ATGGCGAAGCCGGAGAGGAACCCA 3′	5′ AAGGTCACAAGACCGGCTCGGGCC 3′
*pesticin domain protein*	*Pesticin*	NC_021237.1	5′ ATGTCACGCTACGCGATTGATTTCAGTTTTATC3′	5′ TGATGTTCAAGGGCTGGCCGTCGAGAA 3′
*cytotoxin FitD*	*FitD*	EU400157.2	5′ CGCCAACACCGAGCCACAGCCGGAGG 3′	5′ CGCGTTCAGGCCGTCCACATGCGCCAC 3′
*16s rDNA*	*16S rDNA*	NR_114749.1	5′ TGGGAGGAAGGGCAGTTACCTAATACGTGA 3′	5′ TTCCACCACCCTCTACCATACTCTAGC 3′
*β-Actin*	*β-Actin*	NW_004765946.1	5′ ATGAGGCTCAGAGCAAACGTGGTA 3′	5′ AGTCATCTTCTCGCGATTGGCCTT 3′

## Data Availability

Not applicable.
